# Cytopenias in Autoimmune Liver Diseases—A Review

**DOI:** 10.3390/jcm14051732

**Published:** 2025-03-04

**Authors:** Mohammed Abdulrasak, Ali M. Someili, Mostafa Mohrag

**Affiliations:** 1 Department of Gastroenterology and Nutrition, Skane University Hospital, 214 28 Malmo, Sweden; 2 Department of Clinical Sciences, Lund University, 221 00 Malmo, Sweden; 3 Department of Medicine, Faculty of Medicine, Jazan University, Jazan 45142, Saudi Arabia; ali.someili@medportal.ca (A.M.S.); mmohrag@jazanu.edu.sa (M.M.)

**Keywords:** autoimmune liver disease, autoimmune hepatitis, primary biliary cholangitis, primary sclerosing cholangitis, cytopenias, thrombocytopenia, anemia, leukopenia, liver transplantation, IgG4-related disease

## Abstract

Autoimmune liver diseases (AiLDs), including autoimmune hepatitis (AIH), primary biliary cholangitis (PBC), and primary sclerosing cholangitis (PSC), are immune-mediated conditions associated with significant hepatic and systemic manifestations. Among these, cytopenias—defined as reductions in blood cell counts affecting single or multiple lineages—represent a clinically important, though often under-recognized, complication. Cytopenias in AiLDs arise from diverse mechanisms, including immune-mediated destruction, hypersplenism due to portal hypertension, bone marrow suppression, and nutritional deficiencies. These abnormalities can exacerbate bleeding, infections, or fatigue, complicating the disease course and impacting therapeutic strategies. Immune-mediated cytopenias, such as autoimmune hemolytic anemia (AIHA), immune thrombocytopenic purpura (ITP), and autoimmune neutropenia (AIN), are more frequently associated with AIH, whereas cytopenias in PBC and PSC are largely attributed to hypersplenism. Diagnostic evaluation involves a systematic approach combining clinical history, laboratory testing (e.g., complete blood counts, Coombs tests, and nutritional assessments), imaging studies, and bone marrow evaluation in complex cases. Treatment strategies aim to address the underlying cause of cytopenias, including immunosuppressive therapy for autoimmune mechanisms, beta-blockers or splenectomy for hypersplenism, and supplementation for nutritional deficiencies. Challenges include distinguishing between immune- and hypersplenism-related cytopenias, managing drug-induced cytopenias, and optimizing care in transplant candidates. The recently recognized IgG4-related disease, often mimicking cholestatic AiLDs, adds another layer of complexity, given its association with autoimmune cytopenias and hypersplenism. This review aims to act as a guide for the clinician dealing with patients with AiLDs with respect to the occurrence of cytopenias, with a specific focus on pathophysiology and management of these cytopenias. Furthermore, there need to be enhanced multidisciplinary discussions about those patients between the hematologists and hepatologists, with a maintenance of a high index of suspicion for the rarer causes of cytopenias in AiLDs on the part of the treating physician, and there is a need for further studies to elucidate the mechanisms behind the occurrence of cytopenias in AiLDs.

## 1. Introduction

Autoimmune liver diseases (AiLDs) encompass a group of disorders characterized by immune-mediated damage to liver tissue. These conditions include autoimmune hepatitis (AIH), which primarily targets hepatocytes, as well as primary biliary cholangitis (PBC) and primary sclerosing cholangitis (PSC), both of which predominantly affect the cholangiocytes in the biliary system [1]. These diseases are associated with various extrahepatic manifestations, among which the development of cytopenias, though relatively rare, is well recognized [2,3].

Cytopenias in AiLDs encompass anemia, thrombocytopenia, and leukopenia, which may present as either “monolinear” (affecting a single blood cell lineage) or “multilinear” (affecting multiple lineages) abnormalities [4,5]. The etiologies of these cytopenias are diverse, ranging from autoimmune destruction of blood cells to bone marrow suppression resulting in hematological abnormalities, or portal hypertension leading to hypersplenism [6]. These hematologic aberrations can occur individually or in combination [7], potentially complicating the clinical course of an AiLD by contributing to bleeding, infections, or fatigue [8]. The interplay between hematological abnormalities and AiLDs is complex, involving overlapping autoimmune mechanisms, the cytopenia-inducing effects of medications used to treat AiLD, and complications related to cirrhosis [9].

This review aims to provide a comprehensive overview of cytopenias in AiLDs, with the main focus being the pathophysiology and management strategies of these cytopenias. Special attention is also given to the role of IgG4-related disease (IgG4-RD) in hepatobiliary involvement and its overlap with cytopenias in AiLDs.

## 2. Occurrence and Relevance of Cytopenias in Different AiLDs

Distinct patterns regarding the occurrence of cytopenias are observed in AiLDs. In autoimmune hepatitis (AIH), cytopenias frequently coexist, with rare autoimmune-mediated variants such as autoimmune hemolytic anemia (AIHA) [4], immune-mediated thrombocytopenic purpura (ITP) [10], or autoimmune neutropenia (AIN) [11]. AIHA in the context of AIH has been reported in both younger [12,13] and older individuals [14]. In some cases, this overlap appears to be triggered by preceding viral infections, including SARS-CoV-2 [15], parvovirus B19 [16], hepatitis A [17], and primary varicella zoster infection [18]. On the other hand, anemia of chronic disease or hypersplenism-induced anemia is more commonly observed, particularly when cirrhosis develops [19].

Thrombocytopenia due to ITP is rarely reported in AIH [10,20,21,22,23]. Instead, most cases of thrombocytopenia in AIH arise as complications of portal hypertension resulting in hypersplenism [5,24]. Similarly, leukopenia in AIH is predominantly associated with hypersplenism in the setting of cirrhosis [6], although rare cases of autoimmune-mediated destruction specifically targeting neutrophils [25] or pancytopenia due to myelofibrosis have also been documented [26].

In PBC and PSC, hematological abnormalities largely result from altered portal blood flow into the liver due to portal hypertension [27,28]. Notably, these abnormalities may occur even before the onset of cirrhosis [29,30]. This is attributed to the presinusoidal nature of biliary disease, which causes early impairment of portal blood flow, leading to portal hypertension and its complications [31,32]. A subset of PBC patients may exhibit autoimmune-mediated anemia [33] or thrombocytopenia [34]. However, the majority of hematological abnormalities in PBC are attributed to chronic disease or nutritional deficiencies [35]. Similarly, in PSC, anemia or thrombocytopenia is often associated with inflammatory bowel disease (IBD) [36,37], AIHA [38,39], or nutritional deficiencies due to chronic disease [40].

The impact of these hematological abnormalities on the outcomes of AiLDs is substantial. The severity of underlying liver disease often directly correlates with the degree of cytopenias [41], particularly thrombocytopenia, which is associated with complications of portal hypertension [42,43]. The presence of autoimmune hematological disorders alongside AiLDs may indicate a more severe disease course, reflecting greater immunological dysregulation [44,45]. Furthermore, cytopenias can complicate diagnostic procedures, such as liver biopsies [10,46,47], as well as therapeutic interventions, including liver transplantation [48]. Leukopenia, for instance, predisposes patients to severe infections [6,49], while anemia can exacerbate fatigue and significantly diminish the quality of life for affected individuals [50]. A summary table regarding cytopenias’ associations with different AiLDs is presented in Table 1.

## 3. Pathophysiology of Cytopenias in AiLDs

The development of cytopenias in AiLDs is multifactorial, with contributing mechanisms that include immune-mediated destruction, liver-related causes, and complications associated with AiLD treatments. For instance, autoimmune-mediated destruction can affect any of the hematological cell lines. In AIHA, RBC destruction is mediated by immunoglobulin G (IgG) or immunoglobulin M (IgM) autoantibodies. These autoantibodies bind to RBCs, triggering complement activation or promoting splenic sequestration, ultimately leading to their destruction [51,52,53,54]. In ITP, autoantibodies target platelet glycoproteins, resulting in platelet destruction and sequestration in the spleen [55]. In AIN, autoantibodies target neutrophils, leading to their destruction and functional impairment [56,57,58]. These autoimmune mechanisms are most prominent in patients with underlying autoimmune hepatitis (AIH), reflecting broader immune dysregulation in this condition.

On the other hand, hypersplenism is a common cause of cytopenias in PBC and PSC, mainly due to portal hypertension caused by alterations in portal blood flow leading to splenic enlargement, and resulting in the sequestration of blood cells. This trilinear sequestration in the spleen causes thrombocytopenia, leukopenia, and anemia [59,60,61,62]. Thrombocytopenia is often the first observed cytopenia in the setting of portal hypertension [63] and may be mitigated by interventions targeting portal hypertension, such as beta-blockers [64], transjugular intrahepatic portosystemic shunt (TIPS) procedures [65], or in severe cases, splenectomy [66]. Autoimmune causes of thrombocytopenia have also been documented in PSC [67] and PBC [68], as well as autoimmune hemolysis in both conditions [38,39,69,70,71].

Furthermore, chronic inflammation in AiLD can inhibit bone marrow function, primarily through the production of pro-inflammatory cytokines such as tumor necrosis factor-alpha (TNF-alpha) and interleukin-6 (IL-6), which suppress erythropoiesis [72,73]. Additionally, medications used to treat AiLD, including azathioprine (AZA) [74,75,76,77], methotrexate (MTX) [78,79], mycophenolate mofetil (MMF) [80,81], and, to a lesser extent, tacrolimus (TAC) [82,83], may contribute to bone marrow suppression and further cytopenias. Reduced production of thrombopoietin (TPO) and, to a lesser extent, erythropoietin (EPO), also plays a role in the development of thrombocytopenia and anemia, respectively [84,85].

Dysregulated hepcidin production in AiLD can contribute to anemia of chronic disease. Although hepcidin levels are typically elevated in many liver diseases [86], studies suggest that they may be preferentially reduced in AiLD, indicating the presence of competing mechanisms for cytopenia [87]. Nutritional deficiencies, such as those involving vitamin B12 and folate [88,89,90], as well as fat-soluble vitamin E [91], also contribute to cytopenias. Acquired vitamin K deficiency indirectly exacerbates bleeding diathesis through its impact on the coagulation cascade [92].

Overlap syndromes within the AiLD spectrum, such as PBC-AIH or PSC-AIH, or overlap with other immune disorders, including AIH–systemic lupus erythematosus (SLE) [93,94,95,96] or rheumatoid arthritis–PBC [97,98,99,100,101], present their own unique spectrum of cytopenias. Rare associations between Evans syndrome (overlap of AIHA and ITP) and AiLD have also been reported [44,45,102]. Finally, cytopenias, particularly leukopenias, can also be associated with infections, sepsis, or the use of antimicrobials in treating AiLD-related complications. These factors may further complicate the management of patients with AiLD and should remain an area of focus for clinicians [103,104,105,106,107].

## 4. Cytopenias in Specific AiLDs

Certain cytopenias are more commonly associated with specific AiLDs. For example, AIH is frequently linked to AIHA due to the autoimmune nature of AIH, which leads to the destruction of RBCs [12,13,14,15,16,17,18]. Clinically, patients with AIHA may present with pallor and jaundice [108], symptoms that can often be indistinguishable from a flare of the underlying liver disease [109]. However, the laboratory findings in AIHA are typically characterized by unconjugated hyperbilirubinemia, reticulocytosis, and macrocytosis [110]. AIHA in the setting of AIH is more commonly observed in younger individuals [111] and is frequently associated with preceding viral infections such as SARS-CoV-2, parvovirus B19, hepatitis A, or varicella zoster [15,16,17,18].

Thrombocytopenia in AIH can result from portal hypertension-induced hypersplenism [112] or from immune-mediated mechanisms, such as ITP [10,20,21,22,113]. Leukopenia in AIH is primarily due to hypersplenism, similar to thrombocytopenia [112], but it can also result from treatment-related side effects [74,75,76,77,78,79,80,81,82,83] or AIN [56,57,58]. Interestingly, AIN has been documented in AIH patients who are positive for perinuclear anti-neutrophil cytoplasmic antibodies (p-ANCA) [25,56,57,58,114], suggesting a potential shared pathogenetic mechanism, with the p-ANCA antibody serving as a marker. Autoimmune myelofibrosis has also been reported as a rare complication of AIH, leading to pancytopenia [26,115], although most cases of pancytopenia in AIH are attributed to medication side effects [74,75,76,77,78,79,80,81,82,83].

In cholestatic AILDs, such as PBC and PSC, anemia is relatively common. This is primarily due to chronic inflammation, iron deficiency, and other nutritional deficiencies, including vitamin E deficiency. Anemia in these conditions can also result from portal hypertensive gastropathy or gastrointestinal bleeding from varices [60,88,90,91,92]. In patients with PSC-IBD (inflammatory bowel disease) overlap, blood loss through the gastrointestinal tract and nutritional deficiencies are significant contributors to anemia [116,117]. Thrombocytopenia in PBC and PSC is mainly caused by sequestration of platelets due to portal hypertension, which can occur even before the development of cirrhosis [59,60,61]. Leukopenia in PBC is predominantly attributed to hypersplenism [29,59,60,118], whereas in PSC, it may result from either hypersplenism [119,120,121] or drug-induced causes, particularly when immunosuppressive medications like azathioprine are used to manage concurrent IBD [122,123,124].

## 5. Diagnostic Approach

Cytopenias in AiLDs require a systematic approach taking into consideration the potential for multiple factors causing such cytopenias. As previously mentioned, these may include immune-related destruction, portal hypertension-induced hypersplenism, nutritional deficits, immunosuppression-induced cytopenias, or inflammation-induced causes. Thorough clinical and laboratory assessment is key to diagnosis. Table 2 illustrates a summary of the diagnostic tests utilized for diagnosing cytopenias in AiLDs.

History taking may reveal symptoms consistent with the presence of anemia, which includes shortness of breath, pallor, and fatigue [125]. On the other hand, thrombocytopenia may be signified by easy bruising, mucosal bleeding, or the presence of a petechial rash, while infections may signify the presence of leukopenia [126,127]. A review of the underlying liver function and degree of potential AiLD and/or hepatic decompensation, alongside a drug history including immunosuppressants, is important to reach the diagnosis [128,129]. Physical exam may show signs of liver disease, including jaundice, hepatosplenomegaly, and ascites [130]. Splenomegaly is itself a marker of significant hepatic decompensation and is usually a contributing cause for cytopenias irrespective of underlying hepatic disease [131].

Blood tests constitute the most important pathway leading to an underlying diagnosis [132]. A complete blood count (CBC) is the first step to provide information about the severity and type of cytopenias [133]. A peripheral blood smear will help identify certain defining features associated with certain pathologies, e.g., AIHA being associated with spherocytes [134,135]. Elevated LDH, diminished haptoglobin, reticulocytosis, macrocytosis, and unconjugated hyperbilirubinemia will aid in evaluating hemolytic process, while a direct Coombs test will help in determining underlying AIHA [136]. Iron studies, including serum iron, ferritin, and TIBC (total iron binding capacity), help to differentiate iron deficiency anemia from anemia of chronic disease [137]. B12 and folate levels are essential, especially in the cholestatic AiLDs, to assess for anemia with macrocytosis, especially in a PBC setting [138]. With regard to a thrombocytopenia assessment, the mean platelet volume (MPV) and immature platelet fraction (IPF), which—if both elevated—may signify increased platelet destruction, and are therefore associated with ITP [139,140,141]. If no further blood work leads to a diagnosis, bone marrow aspiration and biopsy may be necessary to look into the bone marrow’s cellularity, the presence of aplastic anemia, and evidence of infiltrative lesions within the bone marrow [142]. This is especially relevant if pancytopenia is present without a discernable peripheral causative process, or if there is an inefficient response to a given treatment or suspicion of a concomitant primary myelodysplastic process [9,142]. However, previous studies have demonstrated that—in patients with underlying liver disease, albeit not specifically AiLDs—the yield is relatively low of performed bone marrow biopsies, and they should therefore be done only after consultation with a hematologist and a after a thorough investigation for peripheral causes of cytopenias is performed [142,143].

Imaging studies aid in evaluating the degree of liver disease and hypersplenism. In the gastroenterology outpatient clinic, abdominal ultrasound will help evaluate liver and spleen size alongside indirect signs of portal hypertension, such as portal vein diameter [144]. Elastography of both the liver and spleen may be useful, especially given the correlation of spleen stiffness with the development of thrombocytopenia [145,146]. More advanced imaging including MRI may be relevant, albeit ultrasonography with the aid of elastography may suffice for the purposes of establishing the cause of cytopenia in AiLD unless complicating factors, such as concomitant portal vein thrombosis, exist [147,148,149].

Subspecialist testing—after hematologist consultation—of the blood for thrombopoietin (TPO) may help in assessing TPO deficiency as a cause of thrombocytopenia [150]. An autoimmune panel to test for antiplatelet and anti-neutrophil antibodies may aid in diagnosing ITP [151] and AIN, respectively [152]. Bone marrow biopsy may be helpful in diagnosing these conditions, with ITP demonstrating megakaryocytosis and AIN demonstrating increased neutrophil precursors in the biopsy, albeit these biopsies may be entirely normal in both ITP and AIN [153,154,155]. Hepcidin may help in diagnosing anemia of chronic disease if elevated [156], albeit low hepcidin has been associated with AiLD [87], whereby this test needs to be interpreted with caution.

**Figure 1:**
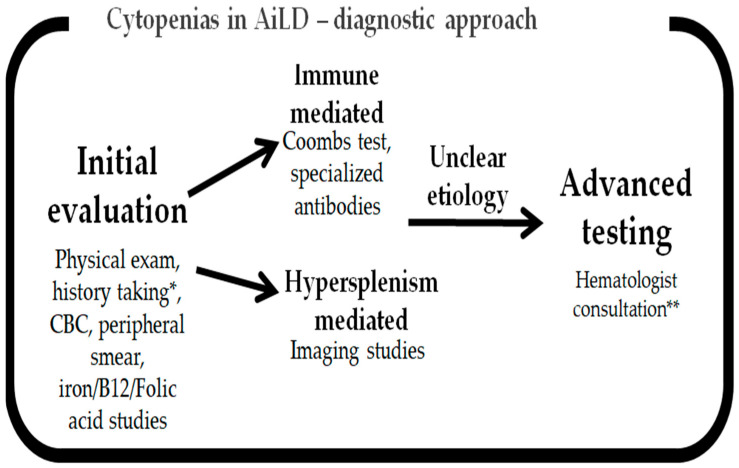
Simplified diagnostic approach for assessing cytopenias in AiLD. CBC—complete blood count. * Involves, amongst other things, medication review, associated autoimmune conditions. ** May involve subspecialized antibody testing, hepcidin levels, TPO levels, bone marrow biopsy.

The main issue with cytopenias in AiLD is distinguishing hypersplenism-related cytopenias from immune-mediated ones. The immune-related cytopenias may have isolated deficits in a single cell line and signs of positive antibodies consistent with AIHA, ITP, or AIN, while hypersplenism can cause a general cytopenia with preferential thrombocytopenia initially due to splenomegaly and portal hypertension. Overlap syndromes may cause multiple concomitant cytopenia mechanisms and, therefore, a careful examination of the presented laboratory and clinical data is required [157,158,159]. Figure 1 provides a visual summary of the diagnostic approach to be considered when assessing cytopenias in AiLDs.

Drug-induced cytopenia is a further challenge, given the association of the majority of the immunosuppressants for AIH with some form of leukopenia. Considering MMF, with its inosine monophosphate dehydrogenase inhibition, lymphopenia is preferentially present and is dose-dependent but reversible if the dose is reduced or discontinued [160,161]. Azathioprine, which also inhibits purine metabolism, causes mainly leukopenia and thrombocytopenia, with this risk reduced if genetic testing is performed prior to its prescription to assess for thiopurine-methyl-transferase (TPMT) activity [162,163,164]. Tacrolimus—with its calcineurin inhibition—is the least likely of the three to cause leukopenia, and should be considered as a treatment for AIH in cases where cytopenias due to MMF or azathioprine occur [165,166]. In all cases of drug-induced cytopenias, it is crucial to establish a detailed timeline that considers the introduction of the drug, the onset of cytopenias, and any changes in dosage or discontinuation, as this aids in identifying causality and guiding appropriate management [167]. Table 3 summarizes the main drugs associated with cytopenias’ development that are relevant for AiLDs.

## 6. Clinical Management of Cytopenias in AILDs

Identifying and treating the underlying etiology is usually the mainstay of therapy in AiLD-associated cytopenias. No specific guidelines for treating cytopenias in AiLDs exist and, therefore, adaptations of existing guidance for similar conditions should be used. For instance, with regard to anemia, identifying the underlying cause, e.g., AIHA, and treating it is of the highest order of importance. AIHA is initially treated using steroids, thus reducing RBC destruction [168]. This is usually initiated if hemoglobin is <7–8 g/dL or if symptomatic anemia occurs. The aforementioned is also the recommended transfusion cut-off [169,170]. If steroids are not sufficient to halt the hemolytic process, second-line agents such as azathioprine or CD20-depleting agents (e.g., Rituximab) may be added [171,172]. In anemia of chronic disease, management of the underlying liver disease is usually sufficient [173]. In iron-deficiency anemia, treating the potential portal hypertensive GI bleeding source is necessary, which may involve iron and RBC transfusions (as in portohypertensive gastropathy), variceal band ligation (if variceal bleed occurs), or TIPS if the pathology is refractory to the aforementioned interventions [174]. Vitamin deficiencies, especially of B12 and folic acid, may coexist in PSC-IBD overlap and should therefore be supplemented [175]. Transfusions should be used in cases of severe symptomatic patients. However, care should be taken to avoid the risk for alloimmunization in patients developing cirrhosis with a potential of undergoing transplantation [176]. This should be done to minimize the risk of organ rejection due to alloimmunization [177,178].

In the cases where thrombocytopenia is dominant, looking for hypersplenism is the best initial step, especially in patients with ensuing cirrhosis or cholestatic AiLDs in pre-cirrhotic stages [179]. This is because mitigating portal hypertension using non-selective beta-blockers (NSBBs) such as Carvdilol or TIPS may aid in minimizing the thrombocytopenia [180]. Splenectomy or splenic arterial embolization may be considered; however, only in rare cases for this specific indication, and after a multidisciplinary discussion including the gastroenterologists and hematologist, where a consideration of underlying conditions on a case-by-case basis is of utmost importance, as guidelines for these specific situations are lacking [181,182,183]. In ITP, usually coexisting with AIH, steroids with the addition of IVIG (intravenous immunoglobulin) may suffice [184]. This is generally started when platelet counts drop below 30 × 10^9^/L, especially when bleeding complications occur [185]. However, Rituximab may be a necessary second-line agent in refractory cases. Splenectomy may be considered in treatment-refractory ITP, while TPO analogues may be used in any of the aforementioned thrombocytopenia types [186,187,188]. Transfusions of platelet concentrate should be undertaken in cases of severe bleeding or prior to invasive interventions, e.g., biopsy, given their association with the development of adverse outcomes [189].

**Figure 2:**
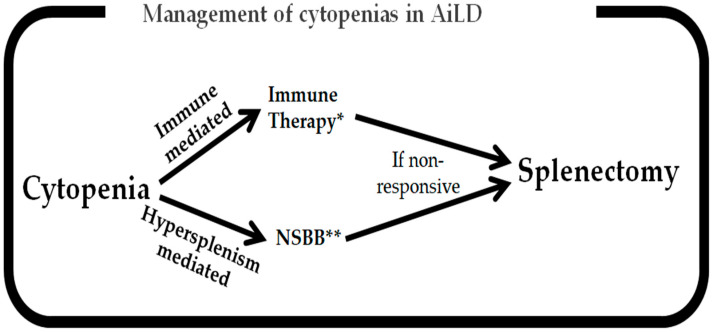
Outline of management strategy for cytopenias in AiLDs. * Immune therapy entails steroids, IVIG, or Rituximab. If refractory to medical treatment, then splenectomy may be required. ** NSBB is first choice; thereafter, portal hypertension remedying interventions such as TIPS/splenic artery embolization and, finally, splenectomy if non-responsive.

In leukopenia, looking for a recent introduction of medications, especially recently introduced immunosuppressants, is key, with dose reduction or discontinuation often being necessary [190]. In AIH, using tacrolimus in these cases where azathioprine or MMF-induced leukopenia occurs is generally safe and provides similar treatment outcomes [191]. In severe neutropenia, G-CSF may be utilized [192], while in AIN, adding steroids or even Rituximab may be necessary [193]. Prophylactic antibiotics, especially if cirrhosis is present, may be necessary, with special care provided to leukopenia associated with these specific agents [194]. General supportive measures for all cytopenias, including nutritional support and actively screening for deficiencies in iron, B12, folic acid, and vitamin K, are essential [195]. Figure 2 outlines the management strategies for the main subtypes of cytopenias in AiLD, stratified by immune-mediated and hypersplenism-mediated cytopenias.

Those awaiting liver transplantation with underlying AiLD are a special group requiring the utmost care of the physicians [178]. This is particularly because thrombocytopenia and underlying coagulopathy may predispose patients to bleeding complications in the perioperative period, which necessitates optimization using platelet transfusion or TPO analogues [196,197]. In the post-transplant period, careful follow-up of the blood counts is essential, with judicious reduction or removal of immunosuppressants or infectious prophylaxis (valganciclovir and/or trimethoprim–sulfamethoxazole) if any cytopenias occur [198,199,200,201]. Multidisciplinary management between hepatology and hematology services is essential to improve outcomes in this particularly complex patient group [202].

## 7. Challenges for the Treating Physician

Multiple challenges in the management of cytopenias in an AiLD setting exist for the practicing physicians, especially the hepatologists. The main challenge is distinguishing the uncommon causes of cytopenias in AiLD from the common ones. The more common ones include anemia of chronic disease and hypersplenism-induced cytopenias; these are well understood and usually identified early on. On the other hand, the autoimmune manifestations, such as AIHA, ITP, or overlap syndromes such as Evans syndrome, are usually underdiagnosed [44,45]. This is mainly due to their insidious onset, rarity, and overlap with the more common causes aforementioned. Therefore, a high index of suspicion of the rarer causes of cytopenia should exist in the repertoire of the hepatologist when managing AiLD patients. Another factor that may cause confusion for the hepatologist is the use of immunosuppression, with its spectrum of hematological side effects. Over-immunosuppression can lead to severe cytopenias with associated infections, while under-immunosuppression may cause disease flares and faster progression to cirrhosis [203]. Therefore, regular follow-up of CBC, alongside maintaining a tight therapeutic drug monitoring, is essential, with switching to alternative immunosuppressants when cytopenias ensue.

In patients who have received a transplant or are awaiting one, the challenge is compounded, with the AiLD itself associated with cytopenias alongside the transplant process itself [204]. This is due to the impact of pre-operative cytopenias (mainly anemia and thrombocytopenia), which can complicate management with increasing bleeding and transfusion risks and risk for renal failure [205,206,207,208]. Hypersplenism is a common cause given the significant portal hypertension usually present pre-transplant [209,210], but the impact of immune-mediated or nutritional causes, as previously detailed, should be properly evaluated and addressed. Post-transplantation cytopenias may worsen due to immunosuppressants, infectious complications, or graft dysfunction [211]. These issues may be complex and necessitate close cooperation, mainly between the transplant hepatologists and the hematologist. A pre-operative TPO analogue may be used to optimize platelet counts pre-operatively [212,213]. However, caution must be maintained given the association with portal vein thrombosis development in certain patients with cirrhosis when TPO analogues are given [214].

## 8. Future Directions and Research Needs

Albeit certain associations between cytopenias and AiLD exist, there remain numerous unresolved issues and opportunities for future research to deepen our understanding of the genetic and immunological basis for the pathogenesis of these cytopenias. For instance, while the contributions of hypersplenism, bone marrow suppression, and immune-mediated destruction are well recognized, little is known regarding the interaction of these mechanisms in the individual patient with AiLD. For example, while IL-6 has a pro-hematopoiesis effect in the physiological state, in the pathological state in other inflammatory conditions (e.g., SLE), it is expected to be inhibitory in action toward the hematopoiesis contributing to cytopenia [215,216]. In addition, the degree of hepatic dysfunction associated with a thrombopoietin synthetic defect needs to be elucidated alongside the detailed pathways, as some studies challenge the notion that TPO itself is the main cause of thrombocytopenia in chronic liver disease [217]. Such future studies may aid in revealing new targets for the treatment of these cytopenias alongside helping in distinguishing between “liver-related cytopenia” and “immune-mediated cytopenias”.

The potential overlaps between AiLD and other systemic conditions, such as SLE, RA, and Sjögren’s syndrome, in some patients are well known [218,219,220]. Knowledge of the mechanisms governing this overlap, however, is lacking. Studies elucidating the genetic and immunological profile of these individuals may help identify specific genetic predispositions, either through HLA haplotypes or immune-regulatory gene polymorphisms, and may in effect aid in identifying patients with susceptibility to developing cytopenias through their AiLD disease course. Given the rarity of both AiLDs and the cytopenias associated with them (especially the autoimmune-mediated ones), multicentric studies with collaborations between hepatologists and hematologists need to occur in this regard in a prospective manner. Such studies may need to include long-term outcomes of patients undergoing TPO treatment for cytopenias, alongside long-term data with regard to the hematological safety profile of the immunosuppressants used for AiLD and for care in the post-transplantation phase in AiLD patients receiving transplants. To add more, the utility of bone marrow biopsies specifically in AiLD patients is not well studied, with the majority of the available studies focusing on the general group of patients with cirrhosis [142]. Such studies may aid in providing more personalized guidelines regarding the management of cytopenias in AiLDs. This lack of specific studies addressing cytopenias in AiLDs is a major limitation of this review, as it is mainly based on inferential data derived from studies where cytopenias in “general” liver diseases are addressed, alongside case reports and smaller case series.

A special mention should be made of the recently discovered entity, namely the multisystemic disorder IgG4-RD, which, albeit not considered a typical AiLD, has its main hepatological manifestation in the biliary tract [221]. This is known as IgG4-RBD [222], and is a condition managed by hepatologists with immunosuppressants, constituted mainly of steroids [223]. The condition may mimic cholestatic AiLDs, especially PSC. In addition, IgG4-RD may overlap with the classic AiLDs [224,225,226] and with the autoimmune-mediated cytopenias [227,228,229] when it involves the biliary tract. In addition, long-term IgG4-RD involving the biliary tract may result in portal hypertension and ensuing hypersplenism [230,231]. Furthermore, bone marrow infiltration of IgG4-positive plasma cells may occur, resulting in cytopenias [232,233]. The distinction between IgG4-RBD and cholestatic AiLDs, especially PSC, is highly relevant given the differing treatment approaches, as, unlike PSC, IgG4-RBD is exquisitely responsive to high-dose steroids [234,235].

## 9. Conclusions

The management of cytopenias in AiLDs is a critical part of hepatology practice, given the relevance of these hematological abnormalities with regard to AiLD treatment, and diagnostic and therapeutic decisions alongside clinical outcomes. The management of these conditions requires a holistic approach and attention to both clinical and laboratory cues, given the multifactorial causes for these cytopenias in AiLD patients. Multidisciplinary management with the close involvement of hematologists is key to success in the management of these complex and often intertwined conditions. Future research on the detailed genetic and immunological background of certain cytopenias in AiLDs may aid in resolving questions and expand the treatment arsenal to include target-specific therapies.

## Figures and Tables

**Table 1:** Summary of different cytopenias and their AiLD associations.

	AiLD	AIH	PBC	PSC
Cytopenia				
Anemia		AIHA, ACD	ACD, vitamin deficiencies	ACD, iron deficiency, PSC-IBD overlap
Thrombocytopenia		Hypersplenism, ITP	Hypersplenism, ITP	Hypersplenism, ITP *
Leukopenia		AIN, hypersplenism, drug-induced leukopenia	Hypersplenism	Hypersplenism, drug-induced *

AiLD—autoimmune liver disease, AIH—autoimmune hepatitis, PBC—primary biliary cholangitis, PSC—primary sclerosing cholangitis, AIHA—autoimmune hemolytic anemia, ACD—anemia of chronic disease, ITP—immune-mediated thrombocytopenic purpura, AIN—autoimmune neutropenia. * Mainly in PSC-IBD overlap.

**Table 2:** Summary of diagnostic tests used to assess for cytopenias in AiLDs.

Specific Test	Diagnostic Utility
CBC	Identifies cytopenia subtype. Anemia → MCV helps define anemia, reticulocyte count identifies bone marrow deficit. Leukopenia → specific cytopenia on differential count. Thrombocytopenia → MPV and IPF aid in assessing size of platelets.
Coombs test	AIHA.
Iron studies, B12/folate studies	Differentiates “deficiency” from anemia of chronic disease.
TPO levels	Thrombocytopenia due to hepatic TPO defect.
Anti-neutrophil antibodies	AIN.
Bone marrow biopsy	Pancytopenia without identifiable cause, unresponsiveness to treatment or suspicion of concomitant hematological disease, e.g., myelodysplasia.

CBC—complete blood count; MCV—mean corpuscular volume; MPV—mean platelet volume; IPF—immature platelet fraction; AIHA—autoimmune hemolytic anemia; TPO—thrombopoietin; AIN—autoimmune neutropenia.

**Table 3:** Summary of drug-induced cytopenias.

	Features	Main Cytopenia	Mechanism	Management *
Associated Drug				
Azathioprine		Leukopenia, thrombocytopenia, anemia	Purine metabolism inhibition	TPMT testing, CBC monitoring, dose adjustment
Mycophenolate Mofetil		Leukopenia	Inosine monophosphate dehydrogenase inhibition	Dose adjustment, CBC monitoring
Tacrolimus		(Rare) leukopenia	Calcineurin inhibition	Dose adjustment, CBC monitoring

CBC—complete blood count. * Signifies the importance of checking other drugs that may interact and cause further cytopenias, such as infectious prophylaxis.

## Data Availability

The data presented in this publication are available within the text of the manuscript.

## Abbreviations

The following abbreviations are used in this manuscript:

AiLD	Autoimmune liver disease
AIH	Autoimmune hepatitis
PBC	Primary biliary cholangitis
PSC	Primary sclerosing cholangitis
AIHA	Autoimmune hemolytic anemia
ITP	Immune-mediated thrombocytopenic purpura
AIN	Autoimmune neutropenia
